# Synergistic Promotion on Tyrosinase Inhibition by Antioxidants

**DOI:** 10.3390/molecules23010106

**Published:** 2018-01-04

**Authors:** Yan Wang, Mi-Mi Hao, Ying Sun, Li-Feng Wang, Hao Wang, Yan-Jun Zhang, Hong-Yan Li, Peng-Wei Zhuang, Zhen Yang

**Affiliations:** 1 Chinese Material Medical College, Tianjin State Key Laboratory of Modern Chinese Medicine, Tianjin University of Traditional Chinese Medicine, Tianjin 300193, China; wangyan@tjutcm.edu.cn (Y.W.); haomimi126@126.com (M.-M.H.); 13920257809@163.com (Y.S.); wanglif930@163.com (L.-F.W.); 13920000655@139.com (H.W.); zyjsunye@163.com (Y.-J.Z.); 2 Tianjin JiaShiTang Technology Co., Ltd., Tianjin 300457, China; lihongyan07@126.com

**Keywords:** tyrosinase inhibitory activity, antioxidant activity, synergistic effect, molecular docking strategy

## Abstract

When exposed to ultraviolet radiation, the human skin produces profuse reactive oxygen species (ROS), which in turn activate a variety of biological responses. Mounting ROS levels activate tyrosinase by mobilizing α-melanocyte-stimulating hormone in the epidermis and finally stimulates the melanocytes to produce melanin. Meanwhile, the Keap1-Nrf2/ARE pathway, which removes ROS, is activated at increased ROS levels, and antioxidant compounds facilitates the dissociation of Nrf2. In this study, we explored the possible suppressing effects of antioxidant compounds and tyrosine inhibitors on melanin formation and the promotory effects of these compounds on ROS scavenging. The antioxidant activity of glabridin (GLA), resveratrol (RES), oxyresveratrol (OXYR), and phenylethylresorcinol (PR) were investigated via the stable free radical 2,2-diphenyl-1-picrylhydrazyl method. The inhibitory effects of the four compounds and their mixtures on tyrosinase were evaluated. l-Tyrosine or 3-(3,4-dihydroxyphenyl)-l-alanine (l-DOPA) was used as a substrate. The results showed that all mixtures did not exhibit synergistic effects with the l-tyrosine as a substrate, suggesting that l-tyrosine is not suitable as a substrate. However, the mixtures of “GLA:RES,” “GLA:OXYR,” “OXYR:RES,” and “PR:RES” demonstrated synergistic effects (CI < 0.9, *p* < 0.05), whereas “GLA:RES” and “PR:OXYR” indicated an additive effect (0.9 ditive1, *p* < 0.05). Furthermore, we used a molecular docking strategy to study the interactions of the four compounds with tyrosinase and l-DOPA. The molecular docking result is consistent with that of the experiment. Finally, we selected RES + OXYR and used PIG1 cells to verify whether OXYR synergistically promotes RES activity on tyrosinase. The two agents had a synergistic inhibitory effect on tyrosinase activity. These results provided a novel synergistic strategy for antioxidants and tyrosinase inhibitors, and this strategy is useful in skin injury treatment.

## 1. Introduction

The human skin is constantly exposed to ultraviolet radiation (UVR), which is an induction factor of reactive oxygen species (ROS). Excessive ROS levels can lead to the formation of pathological skin pigmentation or even direct DNA damage and induce skin injury. Antioxidant defense systems help ameliorate oxidative stress [1,2,3]. Nuclear factor E2-related factor 2 (Nrf2) is a transcription factor that may play a key role in UVR-mediated oxidative stress. Nrf2 regulates several phase II detoxification and antioxidant genes involved in cellular defenses against oxidative stress [4,5].

Skin pigmentation is another mechanism for the prevention of damage due to UVR. Melanin absorbs UVR, thereby protecting skin cells from UVR damage [6]. Therefore, normal skin pigmentation is essential for human health [7]. Tyrosinase (EC 1.14.18.1) is a copper-containing enzyme, widely distributed in fungi, plants, and animals [8]. It catalyzes the pigmentation of skin and is directly related with pigmentation disorders in mammals [9,10]. It is a key target for the discovery and screening of novel inhibitors because of its central role in melanogenesis.

An extremely interesting and delicate relationship exists between antioxidant defense systems and melanogenesis. This relationship is associated with ROS scavenging. The synergistic effect in this relationship increases the effectiveness of antioxidants in scavenging free radicals while tyrosinase inhibitors work, thus reducing melanin production.

Glabridin (GLA) is an isoflavone isolated from the root of *Glycyrrhiza glabra* Linn. It has various pharmacological activities and is capable of inhibiting tyrosinase [11,12]. Resveratrol (RES) is a kind of polyphenolic phytoalexin that is derived from plants. It exhibits broad health benefits, such as antioxidative, anti-inflammatory, and anti-proliferative activities and tyrosinase inhibition [13]. Oxyresveratrol (OXYR) is a powerful inhibitor of tyrosinase and can be used as a skin-whitening and anti-browning agent [14]. Phenylethylresorcinol (PR) is a new skin-whitening agent with strong bioactive ability to inhibit tyrosinase activity [15].

To study inhibitors related to tyrosinase, we evaluated the antioxidant activity and tyrosinase inhibitory activity of four compounds. We found that if these four compounds are in accordance with certain rules, then they can play a synergistic role when combined. This paper is the first written report on this synergistic effect. We also performed molecular docking to study the interactions of each of these compounds with tyrosinase and kelch-like ECH-associated protein 1 (keap1).

## 2. Materials and Methods

### 2.1. Materials and Instruments

GLA (≥95%), RES (≥98%), OXYR (≥98%), and PR (≥98%) are acquired from SABINSA (Sabinsa Corporation, Piscataway, NJ, USA). Free radical 2,2-diphenyl-1-picrylhydrazyl (DPPH) and 25 KU solid tyrosinase from mushroom are all obtained from TCI (Shanghai, China) and Worthington Biochemical (Worthington Biochemical Corporation, Monroe, MI, USA), respectively. Dimethyl sulfoxide (DMSO), 3-(3,4-dihydroxyphenyl)-l-alanine (l-DOPA), and tyrosine are purchased from Sigma Chemical Company (Sigma Aldrich, Munich, Germany). All other chemicals purchased are of high purity and reagent grade. 

### 2.2. Preparation of Antioxidant Solutions

GLA, RES, OXYR, and PR were dissolved and diluted in ethanol.

### 2.3. DPPH Free Radical-Scavenging Capacity Assay

The DPPH free radical-scavenging capacity of the samples are determined according to the method of a previous study [16] with slight modifications. Samples (100l) of various concentrations are added to 100 µL of 0.1 mmol/L DPPH solution in methanol at room temperature. After 30 min, the absorbance is measured at 517 nm with a UV-visible spectrophotometer (MAPADA UV-6100PCS, Shanghai, China). The percentage of free radical (DPPH) scavenging activity (*I*%) is calculated according to following formula:

$$I\%=\frac{OD_{ck}-OD_{sample}}{OD_{ck}}\times 100$$

where *OD_ck_* is the absorbance of the control reaction, which involves all the reagents except the test compound, and *OD_sample_* is the absorbance of the test compound.

### 2.4. Preparation of Individual Solutions and Mixtures

The GLA, RES, OXYR, and PR are dissolved in a mixture of sodium phosphate buffer (PBS, pH = 6.8) and DMSO (80:20), and the prepared solutions are diluted with PBS. Two different individual solutions of GLA, RES, OXYR, and PR are prepared at a ratio of 1:1 [17].

### 2.5. Mushroom Tyrosinase Inhibitory Assay

Inhibitory activity against tyrosinase is examined through an improved spectrophotometric method using l-DOPA and l-tyrosine as substrates. When l-tyrosine is used as the substrate, the reaction mixture is combined with 80 µL of 0.1 mM PBS, 40 µL of 5 mM l-tyrosine [17], and 40 µL of GLA, OXYR, PR, RES, GLA + OXYR, GLA + PR, RES + GLA, RES + OXYR, RES + PR, or OXYR + PR. The reaction mixture is incubated at 37 °C for 15 min. Mushroom tyrosinase (40 µL, 200 units/mL) is added to the reaction mixture and incubated at 30 °C for 20 min.

When l-DOPA is used as the substrate, (1) the reaction mixture is mixed with 80 µL of 0.1 mM PBS, 40 µL of 5 mM l-DOPA, and 40 µL of GLA, OXYR, PR, GLA + OXYR, GLA + PR of OXYR + PR at different concentrations. The reaction mixture is incubated at 37 °C for 15 min. Subsequently, 40 µL of 100 units/mL mushroom tyrosinase is added to the reaction mixtures and incubated at 37 °C for 20 min. (2) The other reaction mixture is mixed with 80 µL of 0.1 mM PBS, 40 µL of mushroom tyrosinase (100 units/mL), and 40 µL of RES, RES + GLA, RES + OXYR, or RES + PR at different concentrations. The reaction mixture is incubated at 37 °C for 15 min. Subsequently, 40 µL of 5 mM l-DOPA is added to the reaction mixture and incubated at 30 °C for 20 min.

Dopachrome formation is monitored at 475 nm with a UV-visible spectrophotometer (MAPADA UV-6100PCS). The rate of the inhibition of tyrosinase activity (*I*%) is determined according to the following formula:

$$I\%=\frac{\left(A_{2}-A_{1}\right)-\left(B_{2}-B_{1}\right)}{\left(A_{2}-A_{1}\right)}\times 100$$

where *A*_1_ and *A*_2_ are the absorbance values of the blank at 475 nm at 0 min and 20 min, respectively, and *B*_1_ and *B*_2_ is the absorbance levels of the test sample at 475 nm at 0 min and 20 min, respectively.

### 2.6. Molecular Docking Strategy

All calculations are performed in Discovery Studio 4.5 client, and the molecular docking studies are computed by CDOCKER module applied in Discovery Studio (Accelrys, San Diego, CA, USA). The structures of GLA, RES, OXYR, and PR are drawn and applied with CHARMm force field and then minimized to the closest local minimum. The crystal structures of tyrosinase (PDB ID: 2Y9X) and keap1 (PDB ID: 4IQK) of *Agaricus bisporus* are downloaded from the Protein Data Bank (http://www.rcsb.org/pdb/home/home.do). Before the docking study, the proteins are prepared by the “prepare protein” module in Discovery Studio 4.5. The ligands are extracted and crystallographic water molecules in the structures are deleted. The configuration and tautomeric states are corrected, and CHARMm force field is applied for minimization.

We calculated the root mean-square deviation (RMSD) between the docked conformation and crystal conformation to estimate the accuracy of CDOCKER. The binding ligands of 2Y9X and 4IQK are contracted and re-docked with receptors. When the molecular docking is completed, the best pose is selected on the basis of binding energy and interactions with active site residues. The best docked pose and co-crystallized conformation are then simply aligned, and the RMSD is calculated. A low value indicates accurate docking method [18].

Subsequently, the drawn compounds are docked to the receptors via the abovementioned method. In this study, three factors, namely, CDOCKER interaction energy, interactions between the compounds and receptor, and compound structures, comprehensively reverse the best pose.

### 2.7. Drug Synergism Analysis of RSE and OXYR in PIG1 Cell

To verify whether the above drugs have a synergistic inhibitory effect on tyrosinase activity in melanocytes, we selected RSE and OXYR for tyrosinase inhibition experiment in PIG1 cells. 

#### 2.7.1. Cell Culture

Immortalized human epidermal melanocyte cells from cell line PIG1 are obtained from Shanghai Guandao Bio-engineering Ltd. Co. (Shanghai, China). PIG1 cells are cultured at 37 °C with 5% CO_2_ in Medium 254 (#M-254-500, Thermo Fisher Scientific, New York, NY, USA) supplemented with human melanocyte growth supplement-2 (Thermo Fisher Scientific, New York, NY, USA) and 5% (*v*/*v*) fetal bovine serum (Thermo Fisher Scientific, New York, NY, USA). 

#### 2.7.2. UVB Irradiation Treatment

The UVB source employed in this study is a Sigma SH4B light therapy device (Shanghai SIGMA High-tech Co., Ltd., Shanghai, China). The energy exposed is measured with a UV304B radiometer equipped with a UV304B sensor (Shenzhen XRC Electronics Co., Ltd., Shenzhen, China). UVB irradiation treatment is conducted when cells reached approximately 70% confluence according to the methods of Zeng et al. [19]. Before UVB irradiation, the supernatant is removed and replaced with PBS. The cells are vertically irradiated with UVB, and the supernatant is immediately replaced with complete medium and RES, OXYR, or RES + OXYR and then incubated at 37 °C with 5% CO_2_. The control group receives the same treatment as the UVB group but is not administered any drug.

#### 2.7.3. Detection of Cell Proliferation Activity by 3-(4,5-Dimethylthiazol-2-yl)-2,5-diphenyl-tetrazolium Bromide (MTT) Assay 

Cell proliferation is detected according to the method of Verma et al. [20]. Cells are plated in 96-well plates at a density of 1 × 10^4^ cells/well in 100 µL of complete medium and then incubated at 37 °C with 5% CO_2_ for 24 h. The culture medium is removed and replaced with PBS. The cells are exposed to UVB irradiation at a dose of 300 mJ/cm^2^. The medium is then immediately replaced with complete medium and RES or OXYR, and the cells are incubated for 24 h. Cells are washed twice with PBS and added to a new culture medium without drugs. Subsequently, 10 µL of 5 mg·mL^−1^ MTT (Gen-view, Calimesa, CA, USA) is added to the culture medium and incubated for 4 h. The supernatant is removed and 100 µL of DMSO (Aladdin, Shanghai, China) is added. Finally, absorbance was measured with an enzyme mark instrument at 490 nm. 

#### 2.7.4. Tyrosinase Activity Assay

Tyrosinase activity is detected by western blotting. PIG1 cells (1 × 10^4^ cells/well) are seeded in 96-well plates and incubated for 24 h in CO_2_ until 70–80% confluence is reached. Subsequently, the complete medium is replaced with 100 µL of PBS, and the cells are exposed to 300 mJ/cm^2^ UVB irradiation. The medium is immediately replaced with complete medium and RES, OXYR, or RES + OXYR and incubated at 37 °C with 5% CO_2_ for 24 h. The intracellular tyrosinase activity of the PIG1 cells are estimated by measuring the l-DOPA oxidation capacity according to the method of Rong et al. [21]. The PIG1 cells are washed twice with PBS, added to 50 µL of 1% Triton X-100, and then incubated at −80 °C for 30 min, allowed to thaw at room temperature, added to 10 µL of freshly prepared l-DOPA (0.25%, Solarbio, Beijing, China), and incubated at 37 °C for 120 min. The products of the reaction are measured by reading the absorbance at 475 nm.

### 2.8. Statistical Analysis

The 50% inhibition concentration (IC_50_) value is determined with SPSS 17.0 statistical software (International Business Machines Corporatio, Armonk, NY, USA). All experiments are performed at least in triplicate. Data are analyzed by unpaired Student’s *t*-test, followed by Dunnett’s multiple comparison test (SPSS version 17.0). P values lower than 0.05 are considered significant. The interactions are calculated using combination index (CI) according to [22]:

$$CI=\frac{{IC}_{50amix}}{IC_{50a}}+\frac{{IC}_{50bmix}}{IC_{50b}}$$

where *IC*_50*a*_ and *IC*_50*b*_ are obtained from pure compounds, and *IC*_50*amix*_ is the concentration of individual compound in the mixture that cause 50% inhibition. Obtained CI values equal, smaller, or greater than 1 indicate an additive, synergistic, or antagonistic effect, respectively [23].

## 3. Results and Discussion

### 3.1. The IC_50_ Values of Antioxidant Solutions

IC_50_ value is the concentration wherein 50% of activity of a compound is lost. The IC_50_ values of GLA, OXYR, PR, and RES solutions are shown in Table 1. GLA, OXYR, PR, and RES are efficient antioxidants, especially OXYR and PR, which demonstrated high free DPPH-scavenging activity. The results are similar to those of a previous report [24]. Meanwhile, GLA exhibited high antioxidant activity. Our results indicated that the capability of the compounds for free radical scavenging are ranked as follows: OXYR > PR > RES > GLA.

### 3.2. The IC_50_ Values of Individual Solutions of Tyrosinase Inhibitory Activities

The IC_50_ values of GLA, OXYR, PR, and RES are shown in Table 2. Our results are consistent with the previous studies on GLA [1] and OXYR [25], which exhibited noncompetitive inhibition of tyrosinase activity with l-DOPA as substrate. RES did not inhibit tyrosinase activity in the l-DOPA substrate but was oxidized before l-tyrosine oxidation. Notably, RES is a *Kcat* or suicide-type inhibitor for tyrosinase [26]. The inhibitory activities of the samples with l-tyrosine and l-DOPA as substrates were ranked as follows: PR > GLA > OXYR > RES and RES > GLA > OXYR > PR, respectively.

### 3.3. Tyrosinase Inhibition and Synergistic Effects of Antioxidant and Tyrosinase Inhibitor

The obtained IC_50_ and CI values of mixtures with l-tyrosine as substrate are summarized in Table 3. All of the mixtures showed an antagonistic effect (CI > 1.1). The obtained IC_50_ and CI values of mixtures with the l-DOPA substrate are shown in Table 4. The CI values showed that the mixtures of “GLA:RES,” “GLA:OXYR,” “OXYR:RES,” and “PR:RES” demonstrated synergistic effects. That is, the CI values were not greater than 1, and the difference between IC_50mix_ and IC_50add_ were significant). The mixtures of “GLA:RES” and “PR:OXYR” exhibited additive effect (0.9 ≤ CI ≤ 1.1).

OXYR had the highest DPPH clearance rate (IC_50_ = 76 ± 0.07 µmol/L). A synergistic effect can be produced when a strong tyrosinase inhibitor are combined, and synergistic effect is positively correlated with the DPPH clearance rate of a strong tyrosinase inhibitor. The DPPH clearance rate of RES (IC_50_ = 142 ± 0.03 µmol/L) was far higher than that of GLA (IC_50_ = 458 ± 0.037 µmol/L), indicating greater synergy in GLA + OXYR than in OXYR + RES.

When PR and OXYR were combined, the DPPH clearance rate (IC_50_ = 119 ± 0.07 µmol/L) and tyrosinase inhibition rate (IC_50_ = 35.5 ± 0.06 µmol/L, using l-DOPA as the substrate) of PR were both less than those of OXYR. Thus, the combination only showed an additive effect.

The DPPH clearance rate of PR was the second highest, whereas the inhibition rate of tyrosinase in the l-DOPA substrate was the lowest, indicating that combining it with either RES or GLA results in a synergistic effect. The synergy of GLA + PR (CI = 0.71 ± 0.04) was greater than that of PR + RES (CI = 0.78 ± 0.02). The CI difference between the two mixtures was small because their DPPH clearance rates were relatively close.

The DPPH clearance rate and tyrosinase inhibition rate of RES (IC_50_ = 2.5 ± 0.28 µmol/L) in the l-DOPA substrate were higher than those of GLA (IC_50_ = 6.0 ± 0.23 µmol/L), indicating the additive effect in the mixture containing RES and GLA.

### 3.4. Molecular Docking Strategy

**Figure 1:**
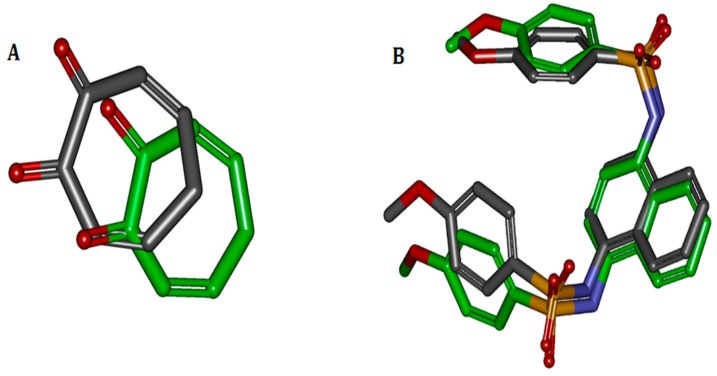
Comparisons between docked and crystal poses. Carbons in green are the crystal poses, whereas the others are docked. Comparison between (**A**) docked conformation and crystal conformation of 2Y9X-OTR with 1.9186 Å RMSD and (**B**) docked and crystal conformations of 4IQK-IQK with 1.0058 Å RMSD.

The binding ligands 2Y9X-OTR, 4IQK-IQK in 2Y9X, and 4IQK were contracted and re-docked to their respective receptors for the calculation of the RMSD. When the docking was completed, the best poses were selected and separately aligned with crystal conformations for the calculation of the RMSD. The results were 1.9186 and 1.0058 Å, indicating that the method effectively reproduced the experimental conditions. The comparisons between the docked poses and crystal ones are shown in Figure 1.

**Figure 2:**
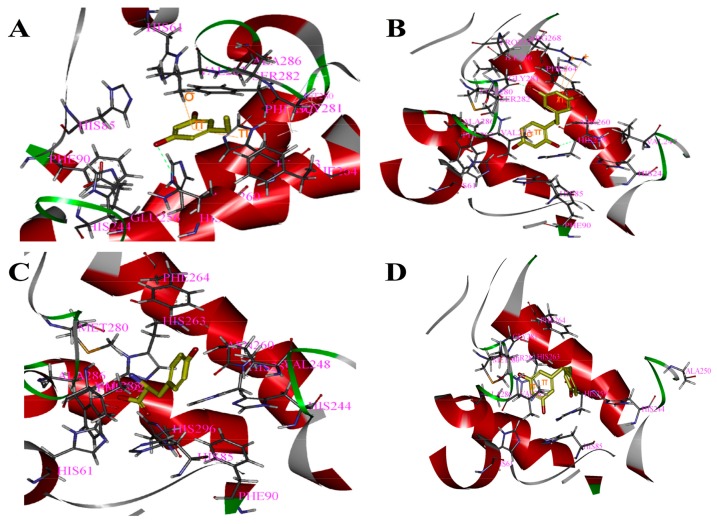
Binding patterns of four compounds (carbons in yellow) in the binding site pocket of 2Y9X. The hydrogen bonds of docked conformation with important amino acids are shown in green dashed line. Interactions between (**A**) PR and 2Y9X; (**B**) OXYR and 2Y9X; (**C**) GLA and 2Y9X; and (**D**) RES and 2Y9X.

**Figure 3:**
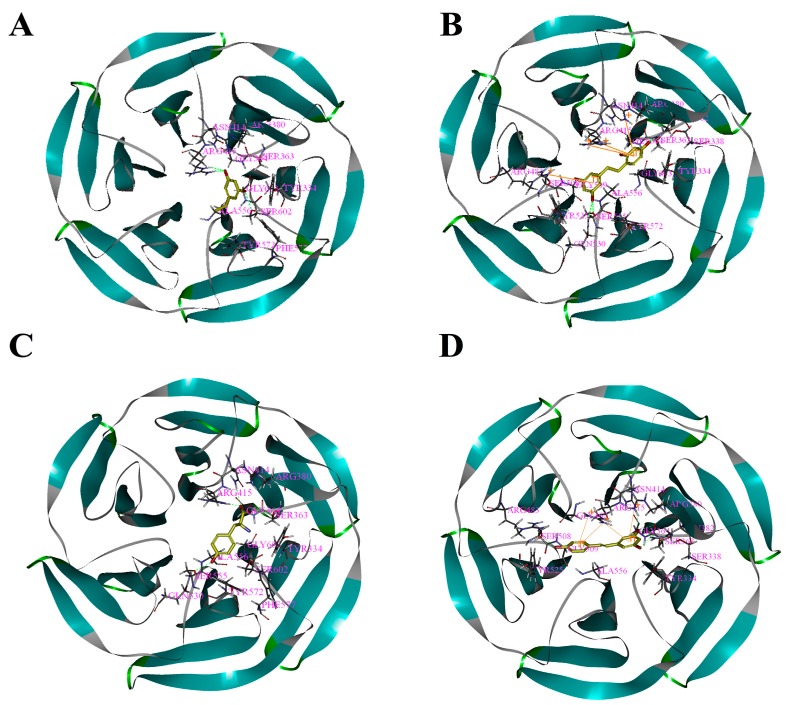
Binding patterns of four compounds (carbons in yellow) in the binding site pocket of 4IQK. The hydrogen bonds of docked conformation with important amino acids are shown in green dashed line. Interactions between (**A**) PR and 4IQK; (**B**) OXYR and 4IQK; (**C**) GLA and 4IQK; and(**D**) RES and 4IQK.

Based on the above study, the four drawn compounds were docked into two receptors, and the results are shown in Table 5. The interactions between the four compounds and receptors are depicted in Figure 2 and Figure 3, respectively. “CDOCKER_INTERACTION ENERGY,” which represents the level of interaction between the compound and the receptor, was calculated during the docking process. A low value reflects good interaction. As illustrated in Table 2 and Table 5, a consistent result was achieved between molecular docking and mushroom tyrosinase inhibitory assay, indicating the good reliability of our results. For the four compounds, the level of molecular docking showed a trend similar to that observed in their effects on tyrosinase, indicating that the two methods can verified by using their results. For PR, the results of molecular docking and antioxidant activity did not show a similar trend. This finding may be related to the algorithm of CDOCKER, which includes the flexibility of the compound and considers the receptor to be rigid. For the other three compounds, the molecular docking and antioxidant activity results exhibited a similar trend, further verifying the reliability of the results.

### 3.5. Drug Synergism Analysis of RSE and OXYR in PIG1 Cell

**Figure 4:**
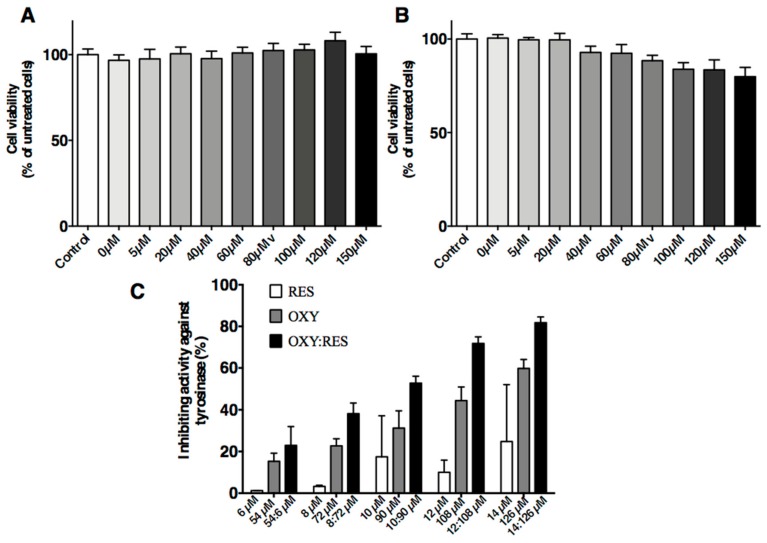
Effects of OXYR (**A**) and RES (**B**) on cell viability after UV irradiation. Dose-dependent inhibition of tyrosinase by OXY, RES and their mixture (**C**). The compounds were incubated with tyrosinase at 37 °C with l-DOPA as substrate. The mixture consisted of OXY and RES (9:1 ratio). The % inhibition was significantly higher in the mixture than the combined inhibition rates of the samples using OXY or RES alone (*p* < 0.01). Each bar shows the mean ± SD of three independent tests.

The MTT assays showed an inhibitory effect on PIG1 cells regardless of UVB dose. UVB doses at 100, 200, and 300 mJ/cm^2^ caused significant effects on the PIG1 cells. However, ≥400 mJ/cm^2^ UVB resulted in high numbers of dead cells. The cell proliferative activity of the 300 mJ/cm^2^ group was 95% of the control group but without statistical significance (*p* = 0.1804; Figure 4A,B). The tyrosinase inhibitory activities of isolated compounds on the l-DOPA substrate were examined.

Each compound was assayed at different concentrations. On the l-DOPA substrate, the mixture containing OXY and RES (9:1 ratio) showed a higher rate of inhibition against tyrosinase activities (IC_50_ = 91.91 µM) than either OXY (IC_50_ = 113.8 µM) or RES alone (IC_50_ > 100 µM). As shown in Figure 4C, the mixture of OXY and RES inhibited tyrosinase activity in a dose-dependent manner. The inhibitory activity was significantly higher in the mixture compared to the sum of the inhibition rate of the samples containing OXY or RES alone (*p* < 0.01). When the concentration of OXY and RES was 90 and 10 µM, the mixture use was 1.68 times higher than that of the sum of the inhibition rate using OXY and RES alone.

## 4. Discussion

Pigmentation is a local color darkening shown on the skin surface, such as melasma and senile plaques. It is caused by UVA ray exposure, which leads to inflammatory reactions, changing the microenvironment of melanocytes in the skin. The above reactions promote melanin production [27,28,29].

Skin types vary according to the concentrations and qualities of eumelanin, which is a major pigment and photo protectant that protects the skin from damage [30]. Natural pigments and melanin in skin and hair seemingly produce endogenous hydrogen. The capacity of eumelanin relies on molecular hydrogen production, which may play a role in protecting skin. To fight against oxidative stress-related disorders, eumelanin pools dihydrogen. This effective pooling must be regarded as a novel element of skin defense system. Eumelanin is usually considered as an absorbent filter or physical barrier of the skin against the penetration of damaging agents [31]. Molecular hydrogen plays major roles in various skin diseases. For instance, it can remove toxic ROS. For dark-skinned population [32], the more melanin-driven hydrogen works, the lower the risk for ROS-mediated skin diseases. Eumelanin forms a skin defense system by producing and pooling molecular hydrogen, and this system acts on toxic agents for the elimination of detrimental effects.

Nrf2, an essential transcription factor for controlling oxidative stress [33], is expressed in human skin tissues, such as human keratinocytes [34,35], melanocyte [4], and human dermal fibroblasts [36]. Cytoprotective antioxidants can be increased by antioxidant compounds through the nuclear accumulation of Nrf2 [36,37]. Some necessary connections seemingly exist between these two protective mechanisms of skin tissues because they both participate in ROS removal.

Tyrosinase is a key factor that limits melanogenesis [9]. Tyrosinase inhibitors are widely used in the cosmetic field owing to their skin-whitening effects [38] and in the food industry because of their inhibitory activity against the enzymatic browning of food products [39]. Unfortunately, the use of tyrosinase inhibitors has potential risks to the human body. For instance, kojic acid, arbutin, and hydroquinone are widely applied as inhibitory agents in the field of cosmetics and known to exhibit strong therapeutic effect. However, they cause serious side effects. Kojic acid and hydroquinone cause genotoxicity and carcinogenesis [40,41,42]. Arbutin is a glycosylated hydroquinone extracted from wheat, pear skins, and blueberry leaves and is easily converted to harmful hydroquinone on the skin surface [42]. Therefore, safe and stable agents are urgently needed. Kojic acid can cause contact dermatitis [43], and rhododendrol can induce vitiligo [44]. Therefore, effort must be devoted for the development of safe and effective tyrosinase inhibitors.

Currently, scientists have been working on the study of natural compounds that offer a direct synergistic effect for tyrosinase inhibition. Research shows that copper ion scavengers and tyrosinase exhibit synergistic effects with tyrosinase inhibitors (non-copper ion scavengers). However, with respect to the CI value, a moderate synergistic effect only exists between the two copper ion scavengers [45]. Moreover, melanin production can be inhibited by noncompetitive and competitive tyrosinase inhibitions in synergy [46].

The sequences of antioxidative activities of GLA, OXYR, PR, and RES and their inhibitory activities against tyrosinase were determined. Our results indicated that tyrosinase activity was greatly inhibited through synergistic between strong antioxidants and strong tyrosinase inhibitors. This synergistic effect was observed only when l-DOPA was used as substrate.

The synergistic effect produced by two compounds was not related to the mode of tyrosinase inhibition, because GLA [1], OXYR [25] and PR were non-competitive inhibitors of the mushroom, whereas RES is a *Kcat* or suicide inhibitor.

Tyrosinase oxidizes phenols to *ortho*-quinones (monooxygenase activity) or catechols to *ortho*-quinones (oxidase activity) in catalytic reactions. It possesses four discrete oxidation states, namely, deoxy-, oxy-, met-, and deact-tyrosinase. The activity of tyrosinase is the first step in melanogenesis, indicating the oxidation of phenols to *ortho*-quinones by oxy-tyrosinase [47,48]. The activity mentioned above also shows the oxidation of bisphenol to quinone at the same time [2,3,5,13,14,18,32,34,37,38,49]. Thus, the activity of tyrosinase reflects the whole oxidation process of phenol to quinone through monooxygenase activity.

## 5. Conclusions

In this study, we demonstrated a novel synergistic compatibility effect among GLA, RES, OXYR, and PR. When the antioxidant activity of compound A was greater than that of compound B, and the inhibition of tyrosinase activity was weaker than compound B. Furthermore, the two compounds were combined in certain proportions. Compound A can increase the inhibition rate of B on tyrosinase, which can produce synergistic effect in vitro when l-DOPA is used as substrate. Finally, we selected RES + OXYR and used PIG1 cell to verify synergistic compatibility and obtained the same result.

## Figures and Tables

**Table 1:** The IC_50_ values of antioxidant solutions.

Samples	IC_50_ (µmol/L)
OXYR	76 ± 0.07
PR	119 ± 0.07
RES	142 ± 0.03
GLA	458 ± 0.037

**Table 2:** Inhibitory effects of single compounds on tyrosinase activity with l-tyrosine or l-DOPA as a substrate.

Substance	IC_50_ (µmol/L)	
	l-Tyrosine	l-DOPA
GLA	1.5 ± 0.03	6.0 ± 0.23
OXYR	2.5 ± 0.06	19.5 ± 0.07
PR	0.28 ± 0.03	35.5 ± 0.06
RES	23.0 ± 0.05	2.5 ± 0.28

**Table 3:** IC_50_ values and CI values of mixtures with l-tyrosine as a substrate.

Compounds	Antioxidant Activity	Tyrosinase Inhibitory Activity	IC_50mix_(µmol/L)	IC_50add_(µmol/L)	CI
PR:GLA	PR > GLA	PR < GLA	16.5 ± 0.04	1.08 ± 0.02 *	2.03 ± 0.02
GLA:RES	GLA < RES	GLA < RES	18.5 ± 0.19	9.98 ± 0.05 *	1.82 ± 0.02
GLA:OXYR	GLA < OXYR	GLA > OXYR	2.5 ± 0.28	2.05 ± 0.07	1.28 ± 0.05
OXYR:RES	OXYR > RES	OXYR < RES	12.5 ± 0.05	9.72 ± 0.05 *	1.36 ± 0.04
PR:OXYR	PR < OXYR	PR < OXYR	2.15 ± 0.03	1.82 ± 0.10	1.18 ± 0.03
PR:RES	PR > RES	PR < RES	17.5 ± 0.28	12.75 ± 0.02 *	1.35 ± 0.05

A significant difference (* *p* < 0.05).

**Table 4:** IC_50_ values and CI values of mixtures with l-DOPA as a substrate.

Compounds	Antioxidant Activity	Tyrosinase Inhibitory Activity	IC_50mix_(µmol/L)	IC_50add_(µmol/L)	CI
PR:GLA	PR > GLA	PR < GLA	18 ± 0.16	23 ± 0.01 *	0.71 ± 0.04
GLA:RES	GLA < RES	GLA < RES	3.5 ± 0.09	3.3 ± 0.17	0.96 ± 0.08
GLA:OXYR	GLA < OXYR	GLA > OXYR	8.1 ± 0.03	15.5 ± 0.05 *	0.57 ± 0.01
OXYR:RES	OXYR > RES	OXYR < RES	2.9 ± 0.16	3.9 ± 0.01 *	0.75 ± 0.05
PR:OXYR	PR < OXYR	PR < OXYR	24 ± 0.03	25 ± 0.02	0.94 ± 0.06
PR:RES	PR > RES	PR < RES	6 ± 0.01	37 ± 0.02 *	0.78 ± 0.02

A significant difference (* *p* < 0.05).

**Table 5:** The results of four compounds docked to two receptors.

CDOCKER_INTERACTION ENERGY (Kcal/mol)	PR	OXYR	GLA	RES
2Y9X	−30.41	−35.77	−36.57	−37.53
4IQK	−20.22	−33.37	−26.35	−30.94
